# LV‐predominant arrhythmogenic cardiomyopathy related to pathogenic DSP‐variant

**DOI:** 10.1002/ccr3.9003

**Published:** 2024-05-31

**Authors:** Soban Ahmad, Husam El Sharu, Robin Fernandes, Mark Kolasa, Constantin Bogdan Marcu

**Affiliations:** ^1^ Department of Internal Medicine East Carolina University Greenville North Carolina USA; ^2^ Department of Cardiovascular Medicine East Carolina University Greenville North Carolina USA

**Keywords:** arrhythmia, cardiac magnetic resonance imaging (CMR), cardiomyopathy, genetics

## Abstract

**Key Clinical Message:**

In contrast to previously thought, arrhythmogenic cardiomyopathy can occur exclusively in the left ventricle in association with autosomal dominant mutation, even without any skin manifestations.

**Abstract:**

We present a case of a 43‐year‐old male with left ventricle (LV)‐predominant arrhythmogenic cardiomyopathy (ACM) caused by a novel p.Q1830 mutation in the desmoplakin (DSP) gene. The patient had a significant family history of sudden cardiac death (SCD) and presented with presyncope and exertional dyspnea. The patient's electrocardiography (ECG) showed frequent premature ventricular complexes (PVCs) with bigeminy and couplet patterns. Cardiac magnetic resonance imaging (CMR) revealed late gadolinium enhancement of the left ventricle (LV) and ventricular systolic dysfunction, suggesting LV‐predominant arrhythmogenic cardiomyopathy. The patient was started on guideline‐directed medical therapy (GDMT), and an implantable cardioverter‐defibrillator (ICD) was implanted for primary prevention. The patient reported significant improvement in his heart failure symptoms at the 2‐year follow‐up. The article highlights the importance of timely diagnosis with multimodality imaging and genetic testing and management of the rare DSP‐related LV‐predominant ACM associated with a high risk of SCD.

## Graphical abstract

Cardiac MRI Half‐Fourier single‐shot turbo spin‐echo (HASTE) axial sequence showing increased signal intensity along the right ventricular free wall (red arrow), interventricular septum (white arrow), and epicardial lateral left ventricular wall (blue arrow). Figure B: Cardiac MRI 4 chamber balanced steady‐state free precession (bSSFP) scout showing chemical artifact on right ventricular free wall (white arrow), interventricular septum (blue arrow), and epicardial lateral left ventricular wall (red arrow).

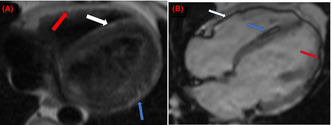

## 1 INTRODUCTION

Sudden cardiac death (SCD) is an unexpected death within 1 h of a witnessed cardiovascular event without any terminal disease or significant trauma.^1^ Approximately 180,000–300,000 people experience SCD in the United States annually, predominantly affecting older men.^2^, ^3^ SCD is primarily associated with arrhythmias, secondary to undetected structural or primary conduction abnormalities. The two most common causes of primary arrhythmias leading to SCD are hypertrophic and arrhythmogenic cardiomyopathy (ACM).^4^

ACM is a group of hereditary disorders that accounts for 20%–25% of SCD cases.^5^ ACM is caused by different genetic mutations affecting proteins making intercellular junctions, particularly desmosomes, resulting in non‐hypertrophic cardiomyopathy characterized by progressive myocyte loss and ventricular fibrofatty infiltration.^6^, ^7^ Clinical presentation of ACM varies widely from being an asymptomatic carrier to experiencing sudden cardiac arrest. Most patients present with palpitations and syncope secondary to symptomatic arrhythmias.^8^ Historically, ACM was primarily considered to involve the right ventricle and was initially called arrhythmogenic right ventricular cardiomyopathy (ARVC).^8^ The Task Force criteria for diagnosing ACM was initially introduced in 1994 and later revised in 2010; it lacks sensitivity for left‐sided and overlapping variants.^9^ A newly proposed criteria, known as the Padua criteria, was suggested in 2020 to guide the diagnosis of left ventricle (LV)‐predominant and biventricular (Biv) phenotypes. However, these criteria are still under the validation process.^10^

Here, we present a patient with LV‐predominant ACM caused by a novel p.Q1830 mutation in the desmoplakin (DSP) gene, resulting in progressive heart failure and a spectrum of electrophysiological abnormalities.

## 2 CASE PRESENTATION

A 43‐year‐old Asian male with a history of hypertension was admitted to the hospital with intermittent episodes of presyncope and exertional dyspnea associated with palpitations of 1 week duration. He is a healthy, active male and did not experience prior syncopal episodes or falls. His only medication was Amlodipine. There is no significant family history of genetic disease. Social history is remarkable for 3–4 drinks of alcohol over weekends and smoking half a pack of cigarettes daily (4 pack/year) without recreational drug use. No cutaneous abnormalities were observed during the physical examination, including woolly hair or keratotic lesions over the palms and soles.

**FIGURE 1:**
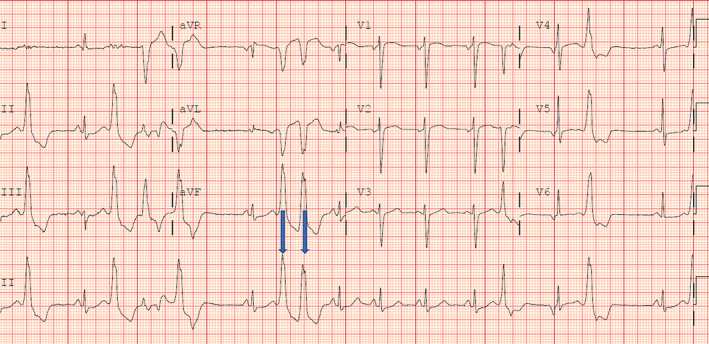
ECG showing sinus rhythm, ventricular bigeminy, and couplet PVC (arrows).

**FIGURE 2:**
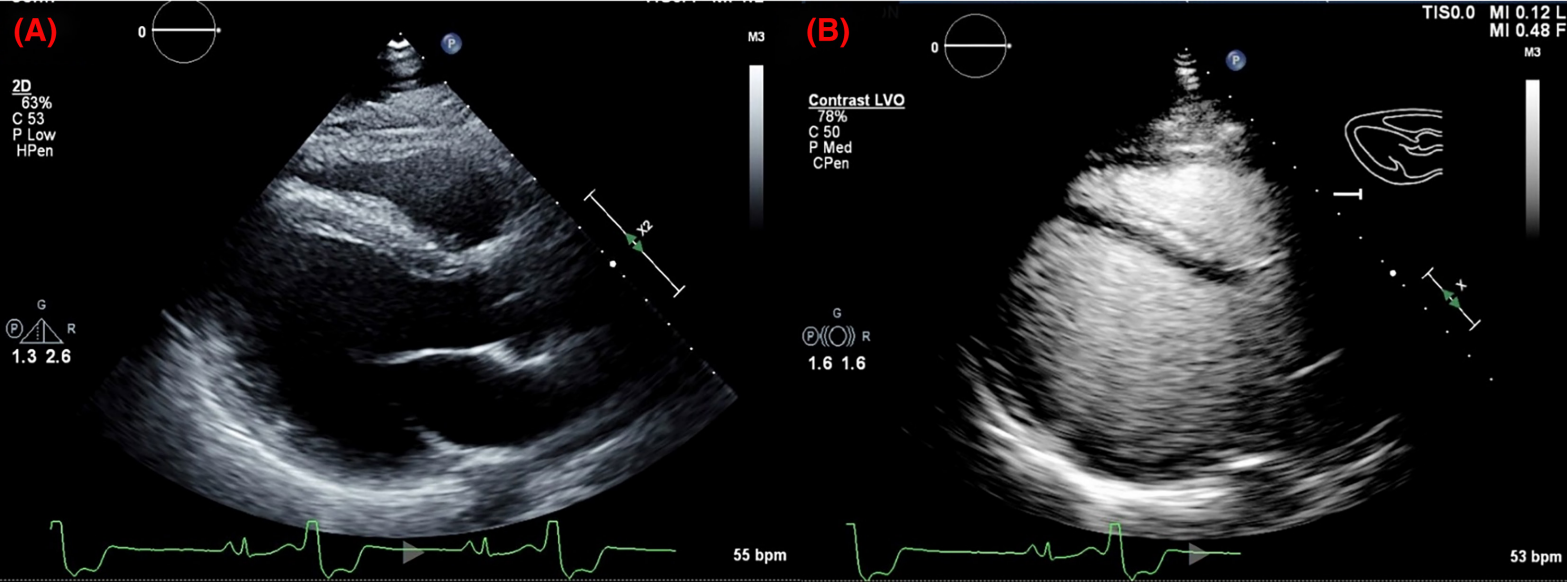
(A) Parasternal long axis view (PLAX) shows a grossly normal right ventricular outflow tract and left ventricle (LV) during mid‐diastole with frequent PVCs captured on the ECG tracing. (B) PLAX view with echo‐enhancing agent shows slightly hyperechoic myocardium on the RV side of interventricular septum (IVS).

Laboratory test results revealed no significant electrolyte abnormalities. Electrocardiography (ECG) showed sinus rhythm (SR) with frequent premature ventricular complexes (PVCs) with bigeminy and couplet patterns (Figure 1). Transthoracic echocardiography revealed a mildly reduced left ventricular ejection fraction (LVEF) of 40%–45% with no significant valvular or structural abnormalities (Figure 2).

**FIGURE 3:**
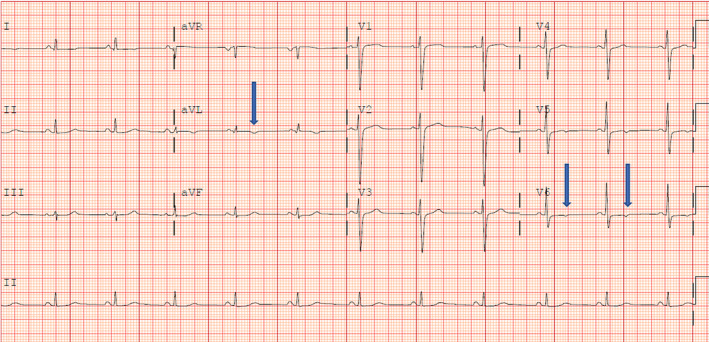
Follow‐up ECG showing low voltage QRS complexes in limb leads and T‐wave inversions in lateral leads (arrows).

The patient was started on metoprolol succinate for a significant PVC burden and was discharged with a close cardiology follow‐up. ECG at follow‐up revealed low‐voltage QRS complexes in limb leads and T‐wave abnormalities in leads I, aVL, V5, and V6 (Figure 3). The patient's 24‐h monitoring at follow‐up showed SR with a 13% PVC burden, bigeminy runs lasting up to 10 min, and brief non‐sustained ventricular tachycardia (NSVT) episodes lasting up to 14 beats.

### 2.1 Differential diagnosis, investigation, and treatment

**FIGURE 4:**
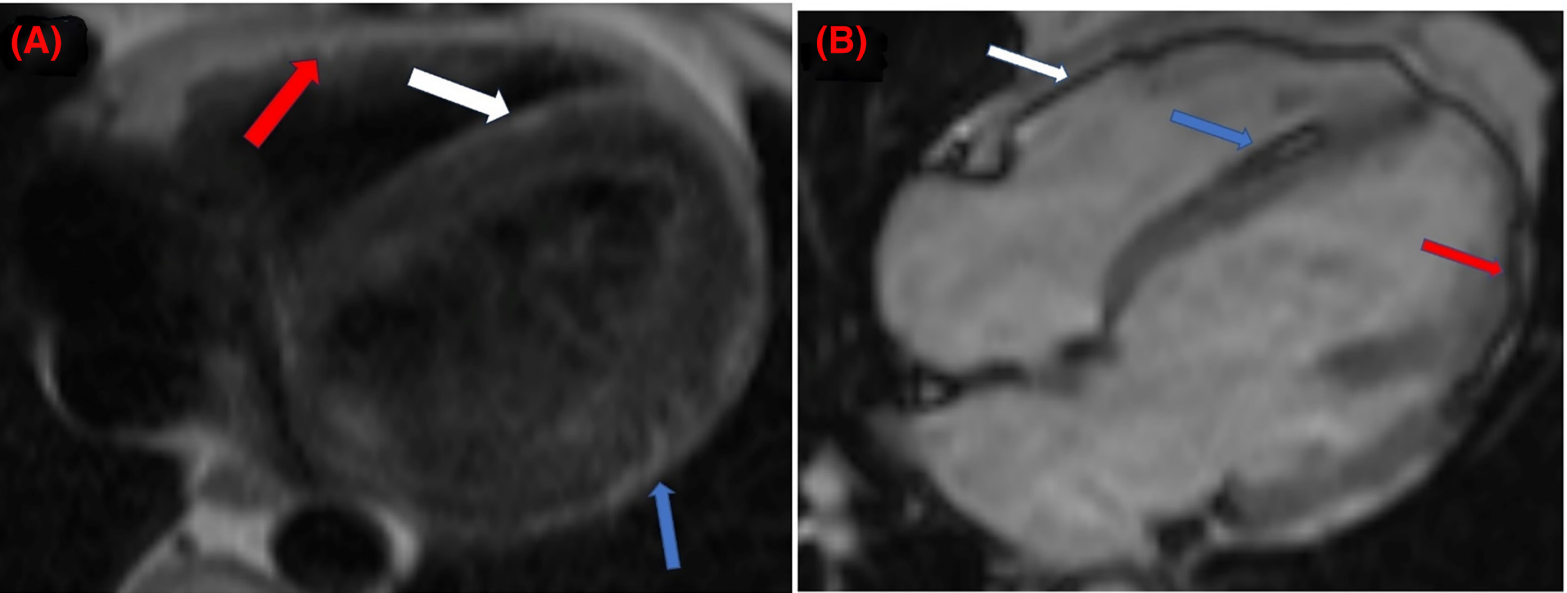
(A) Cardiac MRI half‐fourier single‐shot turbo spin‐echo (HASTE) axial sequence showing increased signal intensity along the right ventricular free wall (red arrow), interventricular septum (white arrow), and epicardial lateral left ventricular wall (blue arrow). (B) Cardiac MRI 4 chamber balanced steady‐state free precession (bSSFP) scout showing chemical artifact on right ventricular free wall (white arrow), interventricular septum (blue arrow), and epicardial lateral left ventricular wall (red arrow).

**FIGURE 5:**
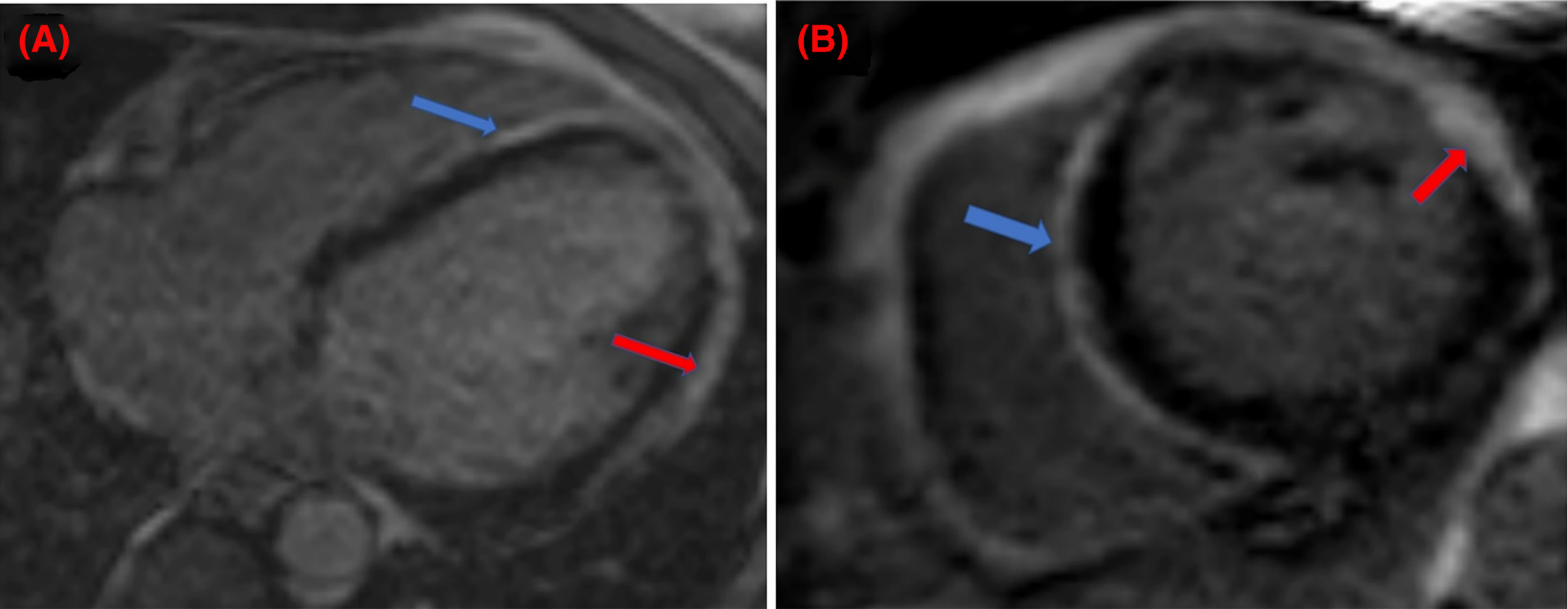
(A) Post‐contrast cardiac MRI axial view showing late‐gadolinium enhancement along the interventricular septum (blue arrow) and lateral left ventricular wall (red arrow). (B) Post‐gadolinium cardiac MRI short‐axial view showing increased signal intensity in the interventricular septum (blue arrow) and left ventricle (red arrow).

As the patient is of young age, has recurrent syncope, significant PVC burden, and new mildly reduced EF, it was thought that the most likely because of his mildly reduced EF. Cardiac magnetic resonance imaging (CMR) was obtained to further identify if there was a structural cause of his PVCs. It showed a mildly dilated left ventricle (LV), a left ventricular end‐diastolic diameter (LVEDD) of 5.2 cm, and mild global hypokinesia with an LVEF of 38%. Pre‐contrast half‐Fourier single‐shot turbo spin‐echo (HASTE) sequence revealed linear regions of increased signal intensity along lateral LV epicardium, right ventricular (RV) free wall, and RV side of the interventricular (IV) septum along with corresponding chemical shift artifact on balanced steady‐state free precession (bSSFP) sequences (Figure 4A,B). Post‐contrast late‐gadolinium enhancement was noted in the same regions consistent with lipomatous replacement (Figure 5A,B). The right ventricle was normal in size and function. Overall, the CMR findings suggested arrhythmogenic cardiomyopathy with predominant LV involvement.

To further identify if his case is associated with a genetic disease an Ambry Genetics Genetic testing was performed with CardioNext, which analyzed 92 genes associated with inherited cardiomyopathies. The patient was found to have a nonsense autosomal dominant pathogenic mutation in the desmoplakin gene described as NM_004415.4(DSP): c.5488C>T (p.Gln1830Ter).

Guideline‐directed medical therapy (GDMT), including Sacubitril‐Valsartan, Eplerenone, and metoprolol succinate, was initiated. An implantable cardioverter‐defibrillator (ICD) was implanted for primary prevention. The patient had both class IIa and IIb indications for ICD implantation. Also, he has symptoms of presyncope in the setting of having CMR demonstrating LV fibrofatty infiltration with LVEF <50%, heterozygous pathogenic DSP variant, male sex, and presence of >1000 PVCs/24 h.

### 2.2 Outcomes and follow‐up

At a 2‐year follow‐up, the patient reported significantly improving his heart failure. Still, he continued to have significant PVCs (22%) on periodic Holter monitoring and did not experience syncope or ICD shocks. The patient was referred for genetic counseling and genetic testing of first‐degree relatives.

## 3 DISCUSSION

Approximately 87% of autopsy studies in patients with ACM‐related SCD demonstrated the involvement of the LV, but isolated LV involvement was observed in only 17% of the cases.^11^ However, a recent CMR study by Chun et al., 2022 reported LV involvement in up to 68% of patients with ACM and was associated with increased cardiac events.^12^ Most patients with LV‐predominant ACM have late gadolinium enhancement (LGE), suggesting fibrofatty infiltration of the inferior or inferolateral LV regions that manifest as LV systolic dysfunction, low‐voltage QRS complexes in limb leads, and T‐wave abnormalities in inferolateral leads.^13^, ^14^ Our patient exhibited significant PVC burden, low voltage QRS complexes in limb leads, mild LV systolic dysfunction, and LGE predominantly involving interventricular septum and lateral LV epicardium suggestive of lipomatous changes. The same fibrofatty infiltrates also act as substrates for life‐threatening arrhythmia and often require ICD placement without significant LV systolic dysfunction.^15^

ACM is a group of hereditary disorders caused by genetic mutations affecting proteins making intercellular junctions, particularly desmosomes, resulting in non‐hypertrophic cardiomyopathy characterized by progressive myocyte loss and ventricular fibrofatty infiltration.^16^ Historically, most people with ACM presented with a right‐sided‐predominant disease. This led to the initial hypothesis that genes affected by mutations causing ACM might exclusively express in cells of the second heart field origin.^8^ However, to date, the exact pathogenetic mechanism of ACM is unknown. Still, numerous genetic mutations affecting various desmosomal proteins, such as plakophilin, desmoplakin, desmoglein, and desmocollin, that play a vital role in maintaining the structural integrity of the heart muscle have been discovered.^17^

Clinical presentation of LV‐ACM can vary greatly, ranging from asymptomatic carriers to those with heart failure and sudden cardiac arrest. Palpitations and syncope due to arrhythmias are usually the most common symptoms.^10^ In 1998, Luis Carvajal‐Huerta reported a constellation of features, including dilated cardiomyopathy, woolly hair, and keratoderma of palms and soles.^18^ Later, Norgett et al., 2000 reported the first human recessive mutation of the DSP gene that caused Carvajal syndrome.^19^ Later, autosomal dominant pathogenic variants of the DSP gene were reported that were not associated with the extracardiac manifestations.^20^ In addition, a recent gene‐centered analysis showed that DSP‐induced ACM involved the LV in almost all cases. It concluded that the most sensitive signs of DSP‐induced ACM are LV systolic dysfunction, LV fibrosis on CMR, and frequent PVCs, which was evident in our patient, who had a heterozygous DSP gene mutation with predominantly left ventricular involvement.^21^ Our patient had a heterozygous, autosomal dominant pathogenic mutation (p.Q1830) in exon 24 of the DSP gene that results in a C to T substitution at nucleotide position 5488 and replaces the amino acid glutamine with a premature stop codon (nonsense mutation) truncating the last 36% of the protein.

Most patients with LV‐ACM have low‐voltage QRS complexes in limb leads, T‐wave abnormalities in inferolateral leads, and LV systolic dysfunction on echocardiography.^13^ CMR often shows late gadolinium enhancement (LGE), suggesting fibrofatty infiltration of the inferior or inferolateral LV regions and reduced LVEF.4 The same fibrofatty infiltrates also act as substrates for life‐threatening arrhythmia. The Task Force criteria introduced in 1994 and revised in 2010 for diagnosing ACM had limited sensitivity for LV‐ACM and overlapping variants.^9^ A newly proposed consensus document (Padua criteria) was introduced in 2020 by international experts to provide a framework to improve the accuracy of diagnosing LV‐ACM. However, further validation is still in progress.^10^

There is no data about the specific guidelines for treating DSP‐related LV‐ACM.^13^ Patients with DSP‐related LV‐ACM are treated based on their cardiac manifestations, such as heart failure with GDMT and arrhythmias with ablation.^4^ In addition to beta‐blocker therapy and catheter ablation, appropriate ICD placement is the only modality to prevent SCD successfully.^16^ Regarding long‐term prognosis, a recent study by Wang et al. (2022) that followed 91 patients with DSP‐induced ACM over 4 years reported that almost one‐third of patients developed heart failure, and 3.6% required heart transplantation without any mortality.^22^

## 4 CONCLUSION

The DSP‐variant related LV‐predominant arrhythmogenic cardiomyopathy is a rare cause of fatal arrhythmias and SCD. Therefore, patients with suspected ACM should undergo a timely evaluation with multimodality imaging, genetic testing, and assessment for ICD implantation, even without systolic dysfunction.

## AUTHOR CONTRIBUTIONS

**Soban Ahmad:** Conceptualization; project administration; supervision; writing – original draft; writing – review and editing. **Husam El Sharu:** Conceptualization; data curation; writing – original draft; writing – review and editing. **Robin Fernandes:** Supervision; validation; visualization. **Mark Kolasa:** Supervision; validation; visualization. **Constantin Bogdan Marcu:** Supervision; validation; visualization.

## FUNDING INFORMATION

None.

## CONSENT

Written informed consent was obtained from the patient to publish this report in accordance with the journal's patient consent policy.

## Data Availability

All generated and analyzed data for this study are included in the manuscript.
